# Principles of brain plasticity in improving sensorimotor function of the knee and leg in healthy subjects: A double-blind randomized exploratory trial

**DOI:** 10.1186/1471-2474-10-99

**Published:** 2009-08-05

**Authors:** Eva Ageberg, Anders Björkman, Birgitta Rosén, Göran Lundborg, Ewa M Roos

**Affiliations:** 1 Department of Orthopedics, Clinical Sciences Lund, Lund University, Sweden; 2 Department of Hand Surgery, Clinical Sciences Malmö, Lund University, Sweden; 3 Institute of Sports Science and Clinical Biomechanics, University of Southern Denmark, Odense, Denmark

## Abstract

**Background:**

Principles of brain plasticity is used in the treatment of patients with functional limitations to improve sensorimotor function. Training is included in the treatment of knee injury to improve both patient-reported function and sensorimotor function. However, impairment in sensorimotor function often persists despite training. Therefore, it was suggested that training programs need to be more effective to improve sensorimotor function after knee injury. The aim of the current study was to investigate if principles of brain plasticity that have been successfully used on the hand and foot to improve sensorimotor function can be applied on the knee. We hypothesized that temporary anesthesia of the skin area above and below the knee would improve sensorimotor function of the ipsilateral knee and leg.

**Methods:**

In this first double-blind exploratory study, 28 uninjured subjects (mean age 26 years, range 19–34, 50% women) were randomized to temporary local cutaneous application of anesthetic (EMLA^®^) (n = 14) or placebo cream (n = 14). Fifty grams of EMLA, or placebo, was applied on the leg 10 cm above and 10 cm below the center of patella, leaving the area around the knee without cream. Measures of sensory function (perception of touch, vibration sense, knee kinesthesia) and motor function (knee muscle strength, hop test) were assessed before and after 90 minutes of treatment with EMLA or placebo. The paired t-test was used for comparisons within groups and the independent t-test for comparisons between groups. The number of subjects needed was determined by an a priori sample size calculation.

**Results:**

No statistically significant or clinically relevant differences were seen over time (before vs. after) in the measures of sensory or motor functions in the EMLA group or in the placebo group. There were no differences between the groups due to treatment effect (EMLA vs. placebo).

**Conclusion:**

We found no effect of temporary cutaneous anesthesia on sensorimotor function of the ipsilateral knee and leg in uninjured subjects. The principles used in this study remain to be tested in subjects with knee injury.

## Background

Neuromuscular and/or strength training is included in the treatment of knee injury and knee osteoarthritis (OA) to improve both patient-reported function and objective function, such as joint range of motion and sensorimotor (neuromuscular) function. However, impairment in sensorimotor function often persists after knee injury and knee OA despite training [1-3]. It has been suggested that good sensorimotor function is of importance for reducing the risk of knee injury [4,5], for achieving better objective and patient-reported knee function after injury [6,7], and in preventing or slowing the progression of OA [8,2]. Longitudinal, prospective studies show that poor muscle function, such as muscle weakness, is a predictor of OA development [9-11]. In this perspective, treatment leading to improved sensorimotor function would be of value for patients with knee injury or OA in the short and long term.

One of the most interesting questions in neuroscience concerns the manner in which the nervous system can modify its organization and ultimately its function throughout an individual's lifetime based on sensory input, experience, learning and injury[12,13]. This phenomenon is often referred to as brain plasticity [14,15]. Plasticity changes can be divided into rapid and long term plasticity. Rapid changes are typically seen minutes after injury or intervention, and are often based on decreased inhibition. Decreased inhibition increases the receptive field size and enables more neurons to be activated by a specific stimulus. This is sometimes referred to as unmasking of synapses or neural structures. Long-term changes are typically seen weeks or months after an injury or intervention and are based on increase or decrease in synaptic transmission or axonal and dendritic sprouting. Synaptic transmission becomes facilitated in a pathway that is frequently used, while those that lay dormant atrophy. Sprouting can be seen in response to injury or to increased functional demand [16]. Axons at the edges of a lesion send new axonal branches into the damaged area and re-innervate dendrites that have lost their synaptic input. Plasticity changes also include changes in nerve signal amplitude and activation of additional cortical areas [14,15].

The primary motor (M1) and sensory (S1) cortex is organized somatotopically, where different body parts project to different parts of the M1 and S1. The somatotopic map does not represent the body in its actual proportions [17,18]. Instead, larger cortical areas are being assigned to sensitive parts or parts with complex motor demands such as the hands and face [19,20]. The cortical representation of different body parts changes constantly, depending on the pattern of afferent nerve impulses, injury and increased or decreased use [21-23]. For example, the forearm is located next to the hand in the somatotopic map [17,18] and by anaesthetizing the forearm, the cortical hand area can expand over the forearm area [24]. Thus, more nerve cells can be available for the hand, resulting in improved hand function. To utilize the central nervous systems' (CNS) ability to change for therapeutic purposes, guided plasticity [25] is an attractive concept with promising results. The potential for cerebral plasticity is, for example, used in treatment of patients to strengthen or promote CNS functions that are lost or weakened [26].

Temporary cutaneous anesthesia of the volar aspect of the forearm, using an anesthetic cream (EMLA^®^), resulted in improved sensory function of the hand in healthy controls [27]. In a randomized controlled trial (RCT), sensory re-learning training in combination with cutaneous forearm anesthesia improved sensory function of the hand compared with sensory re-learning training and placebo in patients with ulnar or median nerve repair [28]. The participants received treatment twice a week for two consecutive weeks, and the effects lasted 4 weeks after the last EMLA treatment. These results suggest that sensory recovery is enhanced by combining training with temporary anesthesia of adjacent body parts. The long lasting effect indicates that this treatment is clinically useful and relevant.

Recently, the same principle of temporary cutaneous anesthesia as that used for the hand has been applied on the foot in uninjured subjects [29]. In this RCT, improvement in sensory function of the foot was observed after cutaneous anesthesia of the lower leg compared with placebo [29]. To our knowledge, the principle of temporary cutaneous anesthesia in improving sensorimotor function of the knee has not yet been tested.

In this first study of a series of experiments, we included subjects without injury. The aim of the current study was to investigate if the principle of brain plasticity that has been successfully used on the hand [27,28] and foot [29] to improve sensory function, can be applied on the knee. We hypothesized that temporary anesthesia of the skin area above and below the knee would improve sensorimotor function of the ipsilateral knee and leg.

## Methods

### Subjects and randomization

Twenty-eight (14 women) physically active subjects aged 18–35 years were included in this exploratory double-blind RCT. The subjects were students at the Faculty of Medicine, Lund University or staff members at Malmö University Hospital. They were enrolled by one of the researchers. Subject characteristics, including activity level [30] and self-reported outcomes assessed by the Knee injury and Osteoarthritis Outcome Score (KOOS) [31,32], are given in Table 1. Exclusion criteria were a history of major orthopedic lesions, such as knee injury or fracture, and allergic reactions to anesthetic agents. The physical activity and age distribution of the subjects in this study were chosen in order to match patients with ligament injuries in the knee. Individuals with ligament injuries to the knee are usually young, aged 18 to 35 years, and physically active at a moderate to high level [1,3]. The subjects were randomly allocated, using a random number generator, to temporary anesthesia using a local anesthetic cream (EMLA^®^) (EMLA group) or a placebo cream (oil and water emulsion) (placebo group). To ensure an equal number of men and women in each group two computer-generated randomization lists, one for women and one for men, were drawn up by a biostatistician and given to the assessor. The assessor allocated the next available number on entry into the trial, assigning the subjects to treatment/placebo. The Research Ethics committee of Lund University approved the study, and all subjects gave their written informed consent.

**Table 1:** Characteristics of the subjects.

Characteristic	EMLA group (n = 14)	Placebo group (n = 14)
Age (y)^*a*^	27 (4.8)	25 (3.9)
Women (n)	7	7
BMI^*a*^	23.0 (2.4)	24.2 (1.8)
Tegner activity level^*b*^	5.5 (4 – 8)	5 (4 – 8)
KOOS subscales		
Pain	100 (1.6)	98 (3.7)
Symptoms	99 (2.1)	98 (5.2)
ADL	100 (0.8)	100 (0.3)
Sport/Rec	98 (4.7)	99 (3.1)
QOL	97 (6.3)	95 (6.5)

^*a*^Mean (SD), ^*b*^median (quartiles), BMI; body mass index. The Tegner Activity Scale, ranges from 0 to 10, least to hardest strenuous activity for the knee. A Tegner activity level of 4 is equal to recreational sports such as jogging, aerobics, or cross-country skiing and a Tegner activity level of 5 is equal to recreational sports such as orienteering or down-hill skiing.

### Protocol and masking

**Figure 1:**
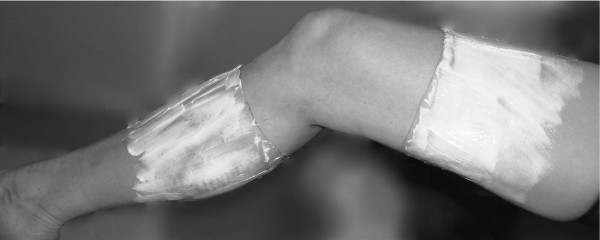
**Application of local anesthetic or placebo cream**. EMLA, or placebo, applied on the leg 10 cm above and 10 cm below the center of patella, leaving the area around the knee without cream.

Fourteen subjects received a local anesthetic cream containing 2.5% lidocaine and 2.5% prilocaine (EMLA^®^, AstraZeneca, Södertälje, Sweden) and 14 subjects received a placebo cream of an oil and water emulsion (DAX, Opus Health Care Inc., Malmö, Sweden). The two creams were identical in color, consistency and packaging. A staff member not participating as an assessor or subject in the study distributed the packages with cream to the assessor. Fifty grams of EMLA, or placebo [29], was applied circumferentially on the leg 10 cm above and 10 cm below the center of patella, leaving the area around the knee without cream (Figure 1). The skin areas where the EMLA/placebo was applied were covered with film wrap and a Tubigrip^® ^stocking (MEDLOCK Medical, Oldham, UK). After 90 minutes, during which time the subject was seated, the EMLA/placebo was carefully washed off. The test leader and the subjects were blinded to group allocation, and the subjects were told not to reveal any possible anesthetic sensation. Therefore, the presence or absence of anesthesia was not verified by the assessor or the subject. The success of blinding was not evaluated.

### Outcome measures

Measures of sensory and motor functions were assessed before and after 90 minutes of treatment with EMLA or placebo. The tests were performed in the order that they are described below. EMLA/placebo was applied and all tests were performed on the right leg only. The assessment took place at the Department of Orthopedics, Malmö University Hospital. An experienced assessor, who was well trained in all outcome measures from previous studies and pilot-testing preceding the present study, performed the measurements.

#### Measures of sensory function

Three measures of sensory function were used; perception of touch, vibration sense and knee kinesthesia. Lower values in these tests indicate better sensory function.

#### Perception of touch

Semmes-Weinstein monofilaments (SWM) were used for assessing perception of touch at the most prominent point of the medial femoral condyle, just proximal of the joint space. Prior to the test, the SWM (nr 4.31, 2.0 g) was demonstrated on the patient's styloid process of the hand, so that the subjects could familiarize themselves with the test. Thereafter, the subjects lay in a supine position and were asked to close their eyes, concentrate on their knee and respond when they felt any sensation of touch. The assessment was performed according to a standardized procedure [33]. Each monofilament, starting with the thinnest and continuing with thicker until response to sensation, was applied perpendicular to the skin for 1.5 seconds and lifted 1.5 seconds. The filament was applied 3 times to the same spot and was bent each time to exert the specific pressure. Feeling the monofilament was recorded when at least one out of three applications was identified by the patient [33].

#### Vibratory perception threshold

Vibratory perception threshold (VPT) was assessed by a biothesiometer (Bio-Medical Instrument, Newbury, OH, USA), according to the manufacturers' manual and previously published methods [34]. Prior to the test, the Biothesiometer was demonstrated on the patient's styloid process of the hand, so that the subjects could familiarize themselves with the test. Thereafter, the subjects lay in a supine position and were asked to close their eyes, concentrate on their foot/knee and respond when they felt any sensation of vibration. The biothesiometer tip was held with uniform pressure at two sites: the most prominent point of the medial malleolus and the medial femoral condyle (same location as that for testing perception of touch). Three consecutive measurements were taken on each site, and the amplitude was replaced to zero between each measurement without moving the biothesiometer tip from the location. The amplitude was increased by 1 Volt per second until the subjects responded to a sensation of vibration. This was noted as the VPT. The first measurement was regarded a trial test, and was, thus, excluded from the analysis. If the difference between the second and third measurement was more than 20%, 2 additional tests were taken. The mean of the second and third, or fourth and fifth, measurements was used in the analysis. High reliability has been reported for the Biothesiometer in healthy subjects [34,35].

#### Knee kinesthesia

Kinesthesia was measured in a specifically designed apparatus, which has been used and described in detail in previous studies, see for example [36,37]. The subjects lay in a lateral decubitus position, were asked to close their eyes, concentrate on their knee and respond when they felt any sensation of movement in their knee. Measurements of the threshold for detection of passive motion (TDPM) were performed towards knee extension (TE) and knee flexion (TF) from the starting position of 20° knee joint flexion, giving the variables TE20 and TF20. The median values of three consecutive measurements of these two variables were determined. The variables from the 20° starting position (TE20 and TF20) have been found to be reliable in uninjured subjects [38]. The sum of TE20 and TF20, giving an index value, was used for statistical analysis.

#### Measures of motor function

Two measures of motor function were used; the one-leg hop test for distance and isokinetic knee muscle strength. Higher values in these tests indicate better motor function.

#### One-leg hop test for distance

The one-leg hop test for distance with the arms free, aiming at a more functional execution of the hop, was used. The one-leg hop test is widely used for predicting functional knee stability [1,3]. Muscle strength, balance and confidence in the knee are contributing factors to the performance of this test. The subjects were told to hop as far as possible, taking off and landing on the same foot, maintaining their balance for about 2–3 seconds. The test was performed three times with each leg, alternating the right and left leg, the hop distance being measured (in cm) from toe in the starting position to heel in the landing position. If the subject improved more than 10 cm between the second and third hop, additional hops were performed until an increase of less than 10 cm was measured. A trial one-leg hop preceded the measurements. The subjects wore shoes, e.g., sneakers. The mean value of the three best hops was used in the analysis. The reliability of this test is high in uninjured subjects [39].

#### Isokinetic knee muscle strength

Measurements of concentric isokinetic strength of the knee muscles were performed with a Biodex Multi-Joint System III isokinetic dynamometer (Biodex Medical Systems Inc., Shirley, New York, NY, USA) with Biodex Advantage software, version 4.0. The standard Biodex knee unit attachment was used. Subjects were placed in an upright position with 90° hip flexion on the Biodex dynamometer chair, and were secured with straps across the chest, pelvis, thigh and ankle. The resistance pad was placed as distally as possible on the tibia while still allowing full dorsiflexion at the ankle. The center of motion of the lever arm was aligned as accurately as possible with the slightly changing flexion-extension axis of the knee joint. The range of motion of the knee joint was set at 5 to 90°. The subjects had their arms crossed over the chest during the test. Standardized verbal instructions and encouragement were given. The subjects were allowed trial tests in order to familiarize themselves with the equipment and the test procedure, before five maximal reciprocal concentric isokinetic knee extensions and flexions at an angular velocity of 60°·s^-1 ^were made. Peak torque/body weight (Nm) was used in the analysis. High test-retest reliability has been reported for isokinetic testing at 60°·s^-1 ^using the Biodex dynamometer [40].

### Statistical analysis

The number of subjects needed was determined by an a priori sample size calculation. No primary outcome measure was determined, since the study has an exploratory character. We expected to find an improvement in more than one of the variables to interpret the results as an effect from treatment. For knee kinesthesia, sample size calculations revealed that at least 12 subjects were needed to detect an improvement by treatment of 30% within groups (SD_diff _0.49), with 80% power at the 5% significance level. For vibration sense, 13 subjects were needed to detect an improvement of 20% (SD_diff _3.3) within groups. For the one-leg hop test, and knee extension peak torque, 2 and 5 subjects, respectively, were needed to detect an improvement by treatment of 10% within groups, with 80% power at the 5% significance level. Based on these sample-size calculations, we included 28 subjects. The paired t-test was used for comparisons within groups and the independent t-test for comparisons between groups. All variables had Shapiro-Wilk statistic of >0.90, except knee kinesthesia. The results were confirmed using non-parametric statistics. Wilcoxon signed rank test, or Mann-Whitney test, was used for ordinal data (perception of touch). Fischer's exact test was used for between-group comparisons in the number of patients with improvement by treatment. Effect size was calculated by taking the difference between the means before and after EMLA/placebo and dividing it by the SD of the same measure before EMLA/placebo [41]. An effect size of <0.50 was considered small, 0.50 to 0.79 moderate, and ≥0.80 large [41]. A level of p ≤ 0.05 was chosen to indicate statistical significance. Group allocation was concealed to the person analyzing the data, until the results were completed.

## Results

No statistically significant or clinically relevant differences were seen over time (before vs. after) in the measures of sensory or motor functions in the EMLA group or in the placebo group. There were no differences between the groups due to treatment effect (EMLA vs. placebo) (Table 2).

**Table 2:** Results for outcomes of sensory and motor functions in the EMLA and placebo groups.

	EMLA group			Placebo group			EMLA vs. placebo
	
	Before Mean (SE)	After Mean (SE)	Mean diff (95% CI) (after minus before)	Before Mean (SE)	After Mean (SE)	Mean diff (95% CI) (after minus before)	Mean diff (95% CI) (EMLA minus placebo)
**Sensory function**							
Vibration sense	11.46	13.11	1.65	10.61	10.96	0.35	1.29
med mall (Volt)	(0.99)	(1.27)	(-0.54, 3.82)	(0.84)	(0.69)	(-0.70, 1.41)	(-1.02, 3.59)
Vibration sense	19.64	21.25	1.61	16.57	17.75	1.18	0.43
med fem cond (Volt)	(1.71)	(1.48)	(-2.12, 5.33)	(1.34)	(1.30)	(-0.79, 3.15)	(-3.58, 4.44)
Knee kinesthesia	2.23	1.95	-0.28	1.88	1.48	-0.40	0.11
(degrees)	(0.38)	(0.45)	(-1.00, 0.43)	(0.19)	(0.11)	(-0.05, -0.73)	(-0.65, 0.86)
Perception of touch	0.04	0.04	p = 0.646	0.07	0.07	p = 0.125	p = 0.265 (before)
(grams)	(0.008, 0.22)	(0.008, 0.16)		(0.04, 0.4)	(0.02, 0.16)		p = 0.769 (after)
							
**Motor function**							
One-leg hop (cm)	134.56	135.21	0.65	144.14	146.05	1.91	-1.25
	(6.85)	(6.31)	(-3.17, 4.48)	(9.47)	(9.79)	(-3.47, 7.28)	(-7.53, 5.03)
Knee ext peak	246.81	239.62	-7.19	252.66	246.73	-5.93	-1.26
torque/body weight (Nm)	(9.90)	(11.10)	(-16.26, 1.89)	(10.40)	(12.89)	(-17.21, 5.35)	(-15.03, 12.52)
Knee flex ext peak	129.56	128.30	-1.26	134.66	136.54	1.88	-3.14
torque/body weight (Nm)	(7.43)	(7.41)	(-6.66, 4.15)	(7.24)	(7.88)	(-3.83, 7.59)	(-10.61, 4.34)

Mean and standard error (SE) and mean difference (95% CI) (after minus before) for the tests of sensory function (vibration sense, kinesthesia) and motor function (one-leg hop test, knee extension and flexion peak torque/body weight) before and after treatment with EMLA/placebo, and mean difference (95% CI) (EMLA minus placebo) between the EMLA and placebo groups (t-test). Median (quartiles) and p-value (Wilcoxon singed rank test, Mann-Whitney test) given for perception of touch (ordinal data).

Med mall = medial malleolus, Med fem cond = medial femoral condyle, Ext = extension, Flex = flexion

### Sensory function before and after treatment with EMLA or placebo

No differences were found between assessments (before vs. after) for perception of touch, vibration sense, or kinesthesia in the EMLA group. No differences were found before vs. after treatment for perception of touch, or vibration sense in the placebo group. A lower value for TDPM, indicating better knee kinesthesia, was found after compared with before treatment in the placebo group (p = 0.026). There were no differences between the groups in effects of treatment for the measures of sensory function (Table 2). The effect sizes were generally small in the EMLA group (between 0.02 and 0.44) and in the placebo group (between 0.11 and 0.56).

### Motor function before and after treatment with EMLA or placebo

No differences were found between assessments (before vs. after) for the one-leg hop test, knee extension or flexion muscle strength in the EMLA group or placebo group. There were no differences between the groups in effects of treatment for the measures of motor function (Table 2). The effect sizes were generally small in the EMLA group (between 0.03 and 0.19) and in the placebo group (between 0.05 and 0.15).

## Discussion

In this first exploratory study on principles of brain plasticity in improving sensorimotor function of the knee, we found no effect of temporary anesthesia of the skin area above and below the knee on sensorimotor function of the ipsilateral knee and leg in uninjured subjects.

Although self-reported and objective function is improved by neuromuscular and/or strength training, it is unclear whether sensorimotor function can be fully restored after knee injury and knee OA. In a recent study, we found that at least one-third of patients with anterior cruciate ligament (ACL) injury or reconstruction had not recovered normal muscle function 2 to 5 years after injury [42]. Possible reasons for this may be that the injury causes a disturbance in the sensory system [43] with possible effects on the central mechanisms and motor response [1], and/or that neuromuscular and strength training programs are not sufficiently effective in improving or restoring sensorimotor function. Moreover, it has been questioned whether training after knee injury can lead to improvement in sensory function although improvement in motor function can be obtained [44,45].

Good sensorimotor function is of importance for the overall outcome after injury [6] and in preventing OA [2,8]. Although improvements are achieved by neuromuscular and/or strength training, impairment in sensorimotor function often persists [1-3]. Thus, it can be argued that training programs need to be more effective in order to improve or restore sensorimotor function after knee injury and knee OA. Hypothetically, the principle of temporary cutaneous anesthesia of adjacent body parts in combination with training, that has been shown to be more effective in improving sensorimotor function of the hand than training only [28], could also be used to improve sensorimotor function of the knee. An advantage is that the selective anesthesia does not affect motor function of the leg. Thus, the individual can use the leg during training while parts of the leg are anesthetized.

The only difference that we found was a lower value for TDPM, indicating better knee kinesthesia, after compared with before treatment in the placebo group. However, the 95% CI was close to zero (Table 2), indicating a small change. Moreover, in a previous study on test-retest reliability, we found that there may be a learning effect in TDPM, shown as a significantly lower value in TDPM on the second test session than on the first test session. In that study, the 95% CI was also quite close to zero and, therefore, we questioned the clinical relevance of this learning effect [38]. Since the 95% CI in the current study was close to zero and our previous study show that there may be a learning effect in TDPM, the clinical relevance of the improvement of 0.40 degrees (95% CI -0.05, -0.73), can be questioned.

There may be several reasons for the lack of effect from temporary cutaneous anesthesia of the skin area above and below the knee on sensorimotor function of the ipsilateral knee and leg in the uninjured subjects in our study. Sensorimotor function may not be impaired in uninjured subjects. Thus, the chance of achieving an improvement in sensorimotor function in these subjects by the short-term intervention that we used is most likely limited. For example, a ceiling effect was noted in some of the measures. The subjects in our study had low values (good sensory function) for both knee kinesthesia and skin sensitivity before EMLA/placebo, limiting the chance of improving these measures by treatment. In addition, the effect sizes were generally small, indicating that the magnitude of change by treatment was small. In previous studies on knee kinesthesia, patients with knee injury have higher values (poorer kinesthesia) than uninjured subjects [44,37]. Thus, the possibility of improving kinesthesia by temporary cutaneous anesthesia may be greater in subjects with knee injury than in uninjured subjects. We tested one site for perception of touch, while several sites were tested in the corresponding study of the foot [29]. In an effort to reduce the ceiling effect, several sites around the knee could be tested in further studies. However, the perception of touch of the knee is not as delicate and discriminative as in the hand or the foot sole. Thus, large effects from temporary cutaneous anesthesia may be needed to detect a change in perception of touch of the knee. Due to the exploratory character of our study, the a priori sample size calculation was based on predictions. A post-hoc sample size calculation estimated that about 30 subjects in each group would be needed to detect improvement in the EMLA group compared with the placebo group for the measures of sensory function and between 5 and 9 subjects in each group for the measures of motor function, with 80% power at the 5% significance level. Thus, the risk of a type II error in the present study cannot be ruled out, implying a need for a larger group of subjects in further studies.

It is well known from animal and human experiments that temporary cutaneous anesthesia of one body part leads to cortical re-organization resulting in a corresponding silent area in the sensory cortex. This allows adjacent nearby body parts to rapidly expand at the expense of the silent cortical area [21,22]. Previous studies on the upper and lower extremity [27-29] as well as the present study have been done on subjects without pain. A peripheral nociceptive stimulus, e.g., a painful knee, is known to induce plasticity changes in the spinal cord and at subcortical and cortical levels. Thus, treating patients with a painful joint using cutaneous deafferentation may give a different result compared to that for individuals without pain. This needs to be addressed in future studies. Neurophysiologic mechanisms in the lower extremity may also differ from those in the upper extremity. Large overlaps in the sensorimotor activation have been shown following movement of the knee, ankle and toes as opposed to the fingers [46]. However, the same plasticity mechanisms likely occur in both the upper and lower extremity, thus making it possible to manipulate plasticity mechanisms also in the lower extremity in order to improve sensorimotor function.

In previous studies on the upper extremity [27,28], the anesthetic cream was applied to the volar aspect of the forearm and in the previous study on the lower extremity the anesthetic cream was applied circumferentially on the lower leg [29]. Based on these previous studies, it would be logical to deafferentate the foot and lower leg in the current study. However, it is very difficult to anesthetize the entire foot using EMLA due to problems with absorption of the EMLA in the sole of the foot and applying an occlusive bandage. Therefore, we decided to anesthetize the skin area adjacent to the knee knowing that following deafferentation, the adjacent cortical areas rapidly occupy the anesthetized area. We also decided to deafferentate circumferentially on the lower extremity because the cortical area devoted to the lower extremity is small compared to the hand and we, therefore, expected that a larger deafferentated skin area was needed (compared to the upper extremity) in order to allow the knee to expand in the primary somatosensory and motor cortex.

We believe that the amount of EMLA that we used (50 grams) and placing of the anesthetic cream (above and below the knee) is adequate in order to expect an increased cortical knee representation. However, the cortical area of the knee is smaller than the cortical area of the hand [17,18]. Thus, larger effects of treatment are needed in order to detect an increase in the cortical area of the knee than in that of the hand. In line with this reasoning, we found no effect of temporary cutaneous anesthesia of adjacent body parts in the measures of sensory or motor functions of the knee in healthy subjects, whereas previous studies reported improvement in sensory function of the hand and foot in healthy subjects after such treatment [27,29]. This could be due to lack of cortical re-organization following the cutaneous anesthesia or that the re-organization was too small to result in a detectable improvement. However, we did not investigate whether the lack of improvement in these measures corresponds to a lack of cortical re-organization. In further studies, neuroimaging methods, such as functional magnetic resonance imaging, can be used to address this question.

Due to the loss of mechanoreceptors after knee injury, the sensory system is disturbed [43], possibly causing effects on sensory and motor functions. Thus, treatment leading to improved sensorimotor function would be of value for patients with knee injury. In line with observations in individuals with hand nerve injury, the sensory deficiency after knee injury can, at least hypothetically, be associated with functional re-organization of the somatosensory cortex of the brain. Thereby, it can be argued that the principle of temporary cutaneous anesthesia in improving sensorimotor function can be used also on the knee. In current neuromuscular training programs for patients with knee injury, principles of brain plasticity such as training of the contralateral extremity are included [1,3]. The aim of these neuromuscular training programs is to enhance unconscious motor responses by stimulating both afferent signals and central mechanisms responsible for dynamic joint control [47,1]. From the present study, we cannot exclude that there is no effect of temporary cutaneous anesthesia of the skin area above and below the knee on sensorimotor function of the ipsilateral knee and leg in uninjured subjects. However, based on the reasoning above, studies on the effect of temporary cutaneous anesthesia for improving sensorimotor function in patients with knee injury and functional limitations are warranted.

## Conclusion

In this exploratory randomized study, we found no effect of temporary cutaneous anesthesia on sensorimotor function of the ipsilateral knee and leg in uninjured subjects. The principles of brain plasticity used in this study remain to be tested in subjects with knee injury and functional limitations.

## Competing interests

The authors declare that they have no competing interests.

## Authors' contributions

EA contributed to the design of the study, was responsible for acquisition, analysis and interpretation of data, drafted and critically revised the manuscript. AB contributed to the design of the study, participated in interpretation of data, assisted in drafting the manuscript, and critically revised the manuscript. BR contributed to the design of the study, participated in interpretation of data, and critically revised the manuscript. GL contributed to the design of the study, participated in interpretation of data, and critically revised the manuscript. ER contributed to the design of the study, participated in acquisition, analysis and interpretation of data, and critically revised the manuscript. All authors read and approved the final version.

## Pre-publication history

The pre-publication history for this paper can be accessed here:

http://www.biomedcentral.com/1471-2474/10/99/prepub
